# Evaluation of the microbial reduction efficacy and perception of use of an ozonized water spray disinfection technology

**DOI:** 10.1038/s41598-022-16953-2

**Published:** 2022-07-29

**Authors:** Luis Alberto Brêda Mascarenhas, Laerte Marlon Conceição dos Santos, Fabricia Oliveira Oliveira, Leticia de Alencar Pereira Rodrigues, Paulo Roberto Freitas Neves, Greta Almeida Fernandes Moreira, Alex Alisson Bandeira Santos, Gabriela Monteiro Lobato, Carlos Nascimento, Marcelo Gerhardt, Bruna Aparecida Souza Machado

**Affiliations:** 1 University Center SENAI/CIMATEC, SENAI Institute of Innovation (ISI) in Health Advanced Systems (CIMATEC ISI SAS), Salvador, Bahia 41650-010 Brazil; 2 University Center SENAI/CIMATEC, SENAI Computational Modeling and Industrial Technology, Salvador, Bahia 41650-010 Brazil; 3 China Three Gorges Corporation–CTG Brazil, Rio Paraná Energia S.A. Rodovia MS-444 s/nº km 58, Ilha Solteira, Selviria, MS Brazil

**Keywords:** Microbiology techniques, Biotechnology

## Abstract

The development of new approaches for the decontamination of surfaces is important to deal with the processes related to exposure to contaminated surfaces. Therefore, was evaluated the efficacy of a disinfection technology using ozonized water (0.7–0.9 ppm of O_3_) on the surfaces of garments and accessories of volunteers, aiming to reduce the spread of microbial pathogens in the workplace and community. A Log_10_ microbial reduction of 1.72–2.40 was observed between the surfaces tested. The microbial reductions remained above 60% on most surfaces, and this indicated that the disinfection technology was effective in microbial log reduction regardless of the type of transport used by the volunteers and/or their respective work activities. In association with the evaluation of efficacy, the analysis of the perception of use (approval percentage of 92.45%) was fundamental to consider this technology as an alternative for use as a protective barrier, in conjunction with other preventive measures against microbiological infections, allowing us to contribute to the availability of proven effective devices against the spread of infectious agents in the environment.

## Introduction

Infectious diseases have emerged and re-emerged over time and such emergencies are motivated by factors inherent to the microbial agent (adaptation and genetic changes, polymicrobial diseases), to the human host (e.g. susceptibility to infection, demography, occupational exposures, inappropriate use of antibiotics) and the human environment (e.g. changing ecosystem, animal populations, lack of public health services, climate and weather)^1^. Although the numbers are not accurate, emerging infectious diseases constitute a substantial fraction of human infections, with profound and lasting effects on society throughout history, shaping the economic, political and social aspects of our civilization. They were responsible for deadly pandemics such as the Bubonic Plague, Severe Acute Respiratory Syndrome (SARS–CoV), swine flu (H1N1) and Ebola^2^.

The recent pandemic caused by SARS-CoV-2 (etiological agent of coronavirus disease 2019-COVID-19)^3,4^ brought about the popularization of the use of disinfection devices to contain the spread of diseases and restore daily life. Sanitization uses mechanical or thermal treatment and the use of biocidal agents to decontaminate body parts, objects or surfaces^5^. Previous studies have demonstrated the benefits of the application of different disinfecting agents^6^ or ultraviolet light devices^7^ for disinfecting hospital environments, portable devices with spray systems for surface decontamination^8,9^, in addition to disinfection chambers with different biocidal agents^10,11^. Spray devices started to be used for the decontamination of inaccessible areas, with the aim of creating a strategy that could improve the cleaning and disinfection of large areas, spaces that are difficult to access or irregular. However, there is a relatively small amount of information regarding this cleaning and disinfection approach^12^. These devices typically rely on electrostatic spray disinfection systems, which transform the disinfectant liquid into aerosols and then apply a charge to each drop so that they are attracted to surfaces by electrostatic forces greater than gravity^13^.

In this scenario, the disinfection tunnels/chambers emerged as a sanitization measure. They can be installed in several places, mainly which can have great circulation of people. The first tunnel was installed in China and was developed by other countries and cities. These portable structures are made of steel and polyvinyl chloride (PVC) with distances ranging from 16 to 25 feet and can be both static and dynamic type. In the static type, the person rotates inside the station for 10–15 s, and disinfectant is sprayed from nozzles arranged around the entire circumference. Dynamic type is a walkway where the person moves 16–25 feet and the device spray the disinfectant all the way through. These tunnels are equipped with infrared detectors (based on sensors) that activate the disinfectant spray whenever a person enters^14^. Basically, these devices spray a mist of a disinfectant solution. However, spraying or nebulizing certain chemicals, such as formaldehyde or quaternary ammonium compounds, usually used as agents in these types of devices, is not recommended by the World Health Organization (WHO) and the Brazilian National Health Surveillance Agency (Anvisa) (Technical Note No. 30/2020), as there is little scientific evidence on the effectiveness of these agents in these technologies, as well as the adverse health effects^15^.

In terms of efficacy evaluations of these devices in use by individuals, few studies address the use of disinfection chambers/tunnels for use by individuals, with the use of different biocidal agents. Mascarenhas et al.^16^ evaluated the microbial reduction capacity of important pathogenic microorganisms in personal protective equipment (PPE) (dressed on a mannequin, mimicking the passage of the device by an individual), using a disinfection chamber with sodium hypochlorite spray to 0.25%. The results showed that in 96.93% of the experimental conditions analyzed, the percentage of reduction was > 99% (the number of viable cells found on the surface ranged from 4.3 × 10^6^ to < 10 CFU/mL). Kampf et al.^17^ also demonstrated that sodium hypochlorite solution at a concentration of 0.1% and 0.5% were effective with a reduction of viral infectivity > 3.0 log10 in 1 min. The ultraviolet rays are known to destroy the DNA of the virus^18^. The radiations from the far-UVC warp the structure of the genetic material of the virus and prevent the viruses from making more copies of themselves. High temperature and high humidity can also reduce and dampen the coronavirus transmission^19,20^. Maurya et al*.*^21^ developed an autonomous tunnel for advanced disinfection of surfaces possibly contaminated by the COVID-19 virus. The technology was used to disinfect clothes and/or open sections in public places, such as airports, schools and shopping malls, in which people passed through the device; and disinfection took place through the application of a disinfectant solution followed by the use for hot air and far-ultraviolet C rays (207–222 nm).

It is important to highlight that the use of agents with a disinfectant action is in itself important for carrying out the disinfection of contaminated surfaces. In nosocomial environments, for example, this action is extremely important and mandatory due to the high exposure to infectious agents in these environments^22^. However, it is also important to keep the need for attention to other places, such as industries and other environments that have a high daily circulation of people^23,24^. Thus, the study on the development of new safe technologies that can be directly applied in high circulation environments, where dissemination easily occurs mainly through the air (via aerosol or droplets), is important and stands out in relation to the choice of the biocidal agent that can be used for these situations. In addition, these studies should be based primarily on the concern about microbial resistance associated with some biocidal agents. The emergence of resistance to disinfectants is a serious threat to life and health safety and to the rational allocation of resources due to the reduced effectiveness of the disinfectant^25^. Then, the choice and use of agents should also be based on the characteristics of the chosen disinfectant, as well as its possibility of use against resistant microorganisms, supporting the development of this type of technologies^26^.

The use of biocidal agents with high antimicrobial activity and low chances of generating microbial resistance, which have a short half-life and decompose into non-toxic molecules, is an effective alternative as a measure of disease control and propagation in these highly disseminated environments. Ozone (O_3_) is among the most powerful oxidants known, with an oxidative potential approximately twice the oxidizing potential of chlorine^27^. The antimicrobial capacity of O_3_ includes not only bacteria, but fungi, viruses and protozoa^28,29^. O_3_ already has documented application in no-touch room decontamination methods because of its powerful antimicrobial properties^30^. It is also one of the biocidal agents with fewer side effects on human health, when dissolved in water (ozonized water), with a multiplicity of applications for dental treatments^31^, and can be used in disinfection devices for human use due to promising evidence of biocompatibility in vitro^32^ and in vivo when compared to traditional disinfectants^27,33,34^.

Therefore, this study aimed to develop, evaluate the effectiveness and perceptive analysis, through the application of questionnaires, of a disinfection technology characterized by a disinfection chamber for instantaneous spraying of ozonized water in the concentration range of 0.7–0.9 ppm of dissolved O_3_ on surfaces of garments and accessories of volunteers, aiming to promote the disinfection of microorganisms originating from environmental contamination on these surfaces and to reduce the spread of microbial pathogens in work and community environments.

## Materials and methods

This study was conducted by recruiting volunteers after approval of the Research Ethics Committee (RES) of the SENAI CIMATEC University Center (Report No. 4.739.411). It is emphasized that this research was carried out in accordance with relevant guidelines/regulations.

**Figure 1:**
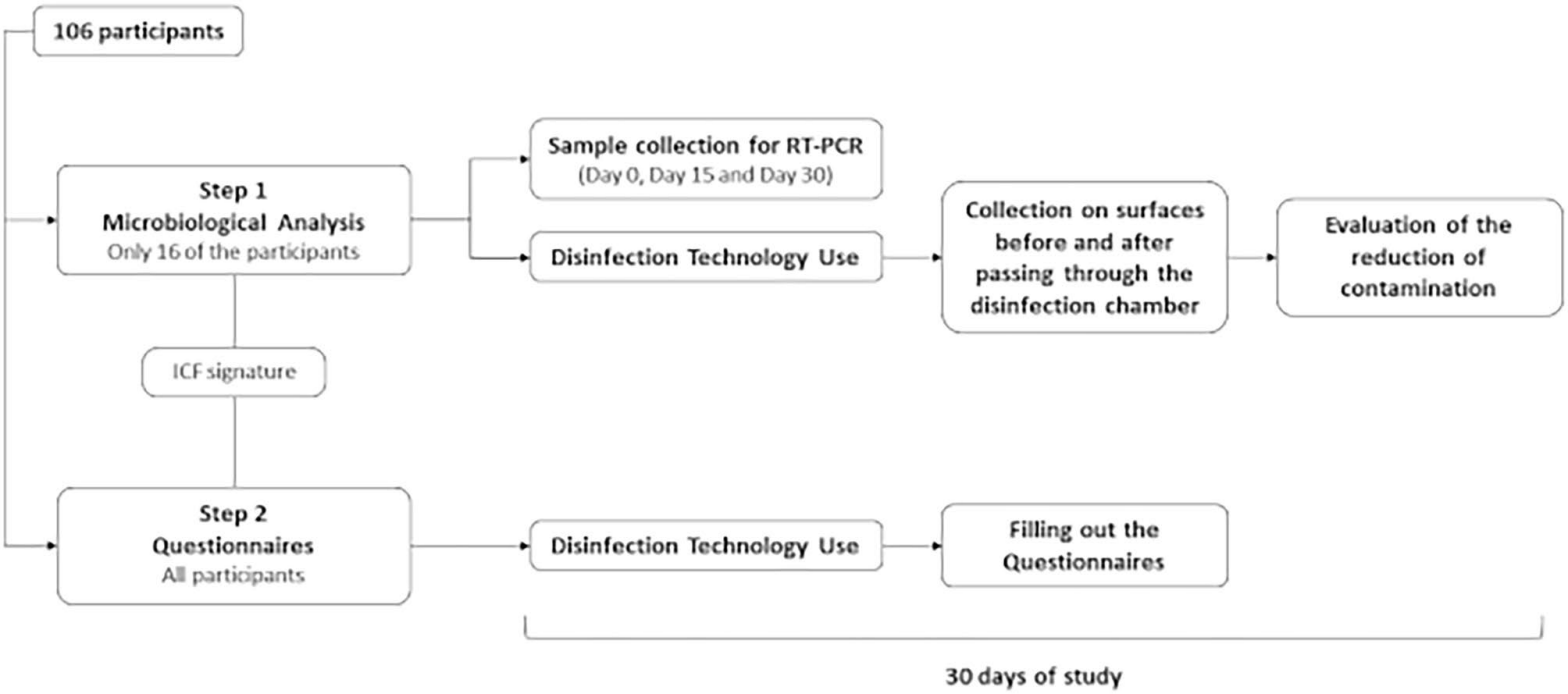
General flowchart of the conduct of this study. The study was divided into two steps that occurred simultaneously for the evaluation of the effectiveness of the ozonized water spray disinfection technology, through microbiological reduction analysis and questionnaire application.

Participants were recruited through the disclosure and completion of a screening form. Those who were selected and volunteered for the study read and signed the Informed Consent Form (ICF). A total of 106 individuals participated in this study, which was divided into two steps that occurred simultaneously, as described in Fig. 1. The first step was to analyze the microbiological reduction efficacy of the disinfection technology (consisting of an ozonation unit and an ozonized water spray disinfection chamber), and the second step was to apply questionnaires to evaluate the perception of use of the disinfection technology. A detailed description of the steps will follow.

### Step 1: Evaluation of the microbiological reduction efficacy promoted by the disinfection technology on participants' garments surfaces

#### Disinfection technology

**Figure 2:**
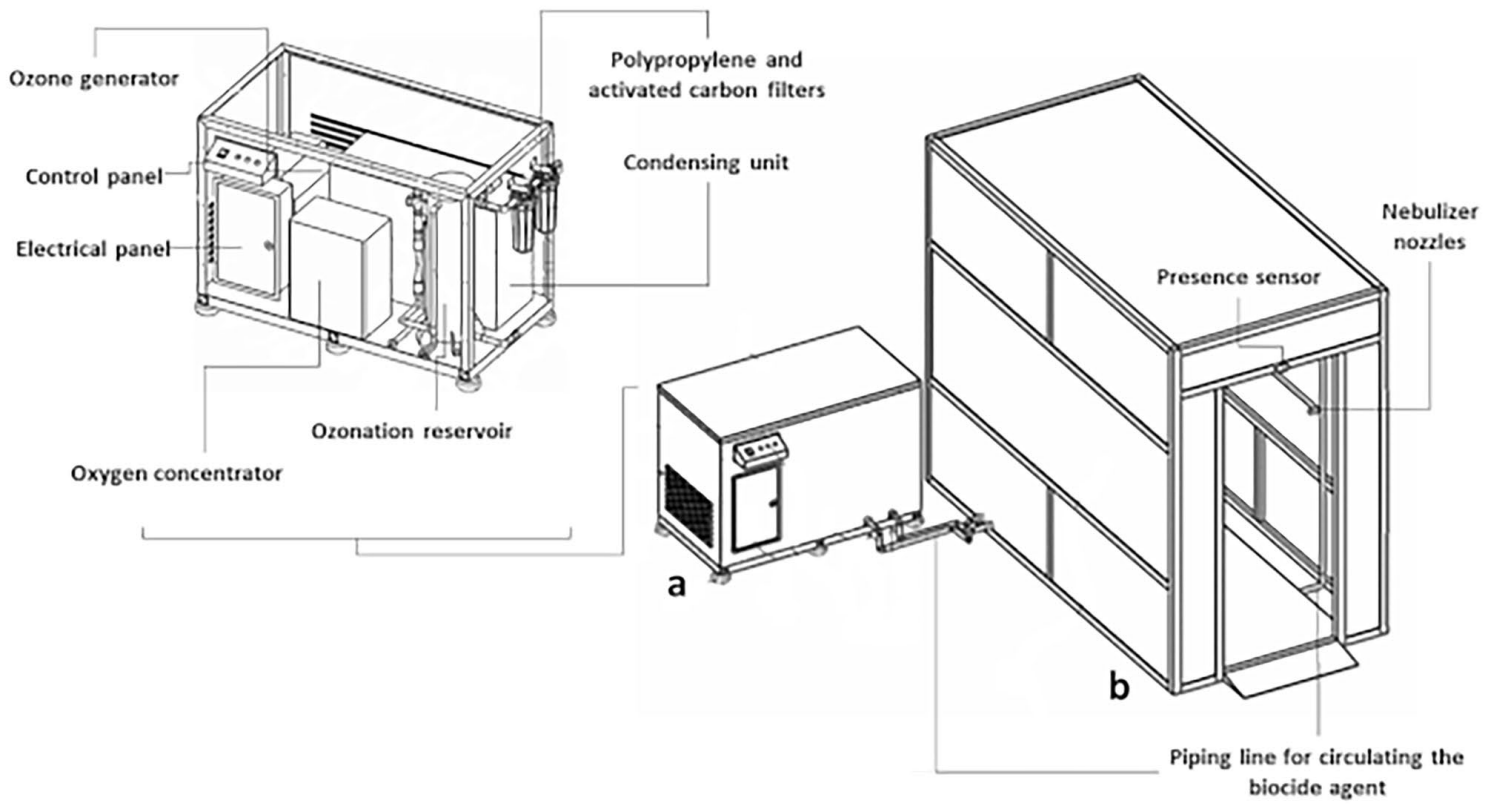
Disinfection technology consisting of (**a**) ozonation unit and (**b**) ozonated water spray disinfection chamber.

The technology has two distinct units (Fig. 2): (1) an ozonation unit, which produces ozonized water under controlled and previously established conditions (temperature of 4–6 °C and O_3_ concentration in water of 0.7–0.9 ppm)^32^. This unit is composed of a metal profile structure, control panel, electrical panel and, inside, an oxygen concentrator with 98% purity output (Yuwell 5LPM), ozone generator (Ozonic model C-20 EL), condensing unit (Elgin ESSE 4130 model) and ozonation reservoir. In the ozonation reservoir, the entire ozonation process, cooling and water supply to be ozonized took place; (2) disinfection chamber for spraying ozonized water, characterized by being a modular structure [dimensions (W × H × D): 1.5 × 2.4 × 3.0 m] composed of a piping line for circulating the biocide agent, presence sensor and nebulizer nozzles. The simulation analysis of the chamber with 12 nozzles used in this study, with high wetting capacity of the exposed area and dispersion of the droplets generated during 30 s, was defined by Neves et al.^35^. The real technology is demonstrated in the Figure S1.

During the time of the study, the system was monitored to verify its correct operation, as well as its ability to maintain the concentration of O_3_ in the ozonized water produced and sprayed into the disinfection chamber. To verify the concentration of the ozonized water released, samples were taken at different times of the day, during the sprinkling time inside the disinfection chamber. The quantification of O_3_ in water was performed using the Spectroquant^®^ Ozone Test 100607 kit (Merck, Kenilworth, New Jersey, USA).

#### Microbiological reduction analysis

The microbiological analyses were performed by evaluating the disinfection process promoted by spraying ozonized water into the disinfection chamber. Of the total 106 participants, 16 agreed to participate in this analysis, and collections for microbiological analysis occurred according to a schedule set by the study team. These 16 participants made use of the disinfection technology over a period of 30 days and, for safety reasons due to the pandemic of COVID-19, samples for RT-PCR (reverse transcription polymerase chain reaction) analysis were collected on Day 0 (screening), Day 15, and Day 30 of the study, in order to ensure the withdrawal of the participant in case of a positive diagnosis for the disease during the study. We already point out that none of these participants were diagnosed with COVID-19 during the study.

**Figure 3:**
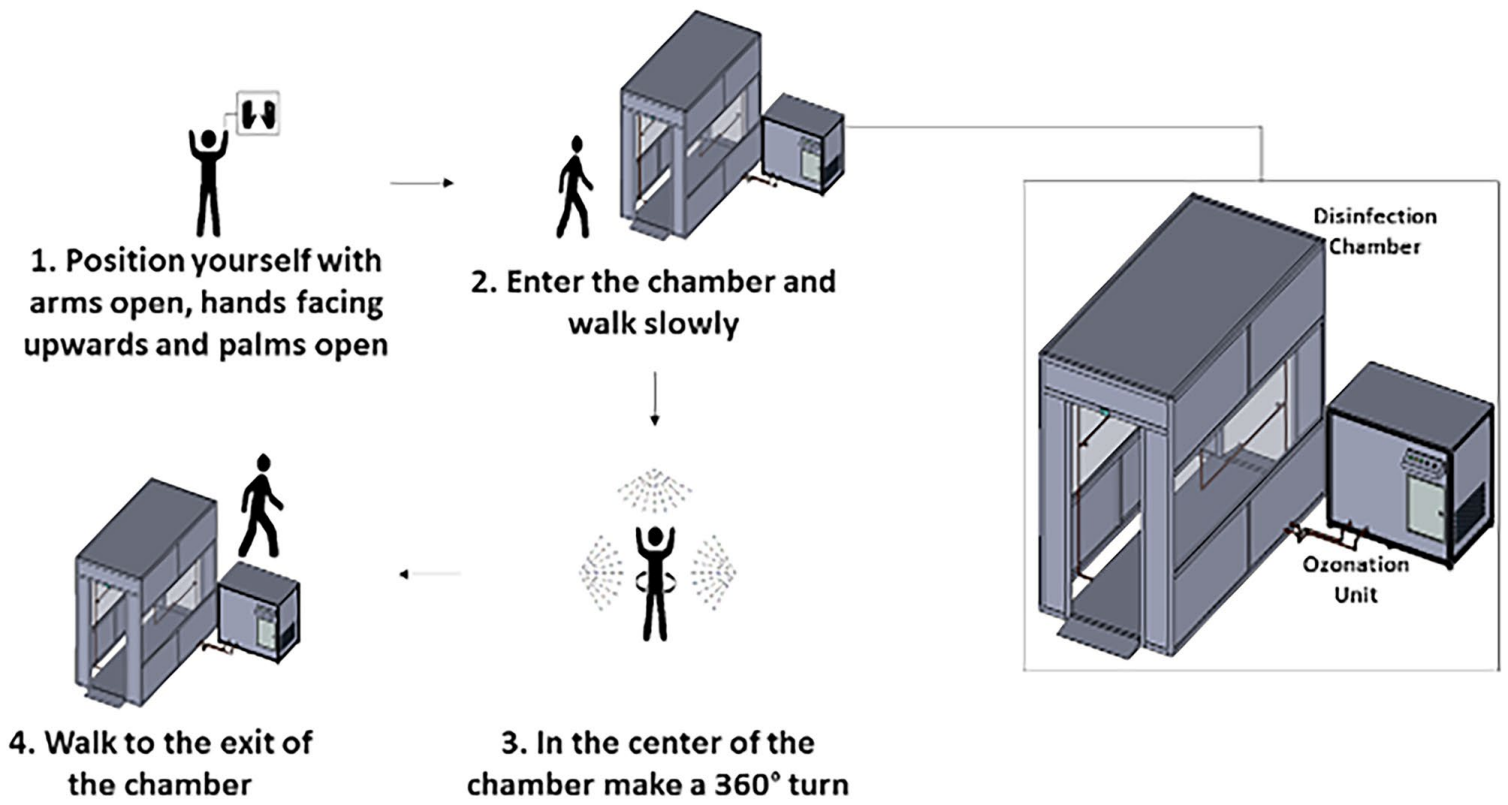
Instructions given to the participants for correct use of the disinfection technology.

The surfaces evaluated were items of garments of the participants: protective mask, shirt, and boots (in some cases, additional accessories were evaluated: glasses, watch, and helmet). The participants were informed about the instructions for use of the device (Fig. 3), and passed for 30 s twice a day, once in the morning (first passage before the beginning of the work day) and once in the afternoon (second passage at the end of the work day).

The collections were performed in demarcated areas (30 cm^2^) of these surfaces before and after the participants had passed through the disinfection chamber, on days previously determined for each of them. While the right side was used for the control, the left side was used for test, according to a study described by Oliveira et al.^36^. The demarcated areas were defined according to experimental tests performed by Neves et al.^35^, where the engineering parameters of the associated disinfection technology were evaluated, as well as the wettability at different points on the surfaces. Thus, the areas chosen for each surface were defined taking into account the same wettability effects between them, not affecting the experimental results.

The collection on these surfaces was performed using a swab immersed in 5 mL of neutralizing solution (SRK Swab Rinse Kit, COPAN Diagnostics) and its content was used to analyze the number of viable cells on the surface for the control and after exposure to the disinfection chamber (a period of one minute was allowed for collection after passing through the chamber).

Then, the swab-immersed samples were vortexed and serial dilutions were performed with inoculations in PCA (plate count agar) for bacteria and SDA (Sabouraud Dextrose Agar) with Chloramphenicol for yeasts, which were cultivated at 37 and 30 °C for 24 and 48 h, respectively. The number of colonies on culture plates was defined in a colony counter after an incubation period of 24 and 48 h. The experiment was carried out in triplicate.

The average values of CFU/cm^2^ were calculated from the formula provided by the neutralizing solution kit used (SRK Swab Rinse Kit, COPAN Diagnostics): (number of colonies × volume of solution × dilution factor (1000))/area of the collection surface = CFU/cm^2^. In this case, the collected surface area was 30 cm^2^. Using the average values of these counts, the microbial reduction percentages was calculated according to the following formula: Microbial reduction percentage (%) = (control CFU − test CFU)/control CFU) × 100. In addition, the logarithmic scale reduction factor (Log_10_) was calculated using the formula RF = Log_10_ (A) − Log_10_ (B), where A is the number of colonies recovered from the unexposed (control) and B is the number of colonies recovered from the exposed (test) to O_3_. Data from two independent experiments were plotted in GraphPad Prism version 8.4.3 (686) (GraphPad Software, San Diego, California, USA, www.graphpad.com).

### Step 2: Evaluation of the perception of the use of disinfection technology through the application of questionnaires

A cross-sectional descriptive study was carried out using a questionnaire to evaluate the perception of the study participants regarding the new disinfection technology developed, as well as their familiarity with ozonized water and their understanding of the product's biocide action, taking into consideration the appearance of any discomfort and the feeling of safety. In addition to the 16 participants from stage 1, other 90 participants (totaling the 106 participants recruited for the study) made use of the disinfection technology on random days and answered the disinfection technology evaluation questionnaire. The questionnaire was performed by qualified researchers of the study. The following questions were asked to the participants:

1. Do you agree that ozonized water can be an alternative for disinfecting materials and surfaces, as it has antimicrobial action?
2. Ozone can be an effective alternative in controlling the spread of disease. Do you agree with this statement?
3. Did you experience any discomfort after using the disinfection chamber?
4. If YES to the previous question, please tick which discomfort(s) you felt (claustrophobia, cough, nausea, headache, lacrimation, upper airway irritation, skin irritation, others).
5. Specify the degree of irritation if you answered the previous question: mild, moderate or severe.
6. After using the disinfection chamber, were the surfaces moistened?
7. Does the disinfection chamber act as an additional barrier in protecting workers in general?
8. Can the use of the disinfection chamber lead to a false sense of security?
9. If you answered “agree” or “strongly agree” to the previous question, why do you think using the camera leads to a false sense of security?
10. Even with the use of the chamber, must all personal hygiene procedures be strictly followed?
11. Can the use of the disinfection chamber with ozonized water be a safe alternative for surface disinfection?
12. If you answered “Disagree” or “Strongly disagree” to the previous question, why don't you find it a safe alternative?
13. Do you have any notes or comments you would like to add about the camera?

### Statistical analysis

Statistical analyzes were performed using the GraphPad Prism version 8.4.3 program (686) (GraphPad Software, San Diego, California, USA, www.graphpad.com). The Shapiro–Wilk test was used to verify data normality for the analyzes performed in this report. A first analysis was carried out to evaluate the effect of passing through the disinfection chamber. For this, the average values ​​of CFU/cm^2^ calculated from the formula provided by the kit of the neutralizing solution used (SRK Swab Rinse Kit, COPAN Diagnostics) were used: (number of colonies × volume of solution × dilution factor (1000))/collection surface area = CFU/cm^2^. In this case, the surface area collected was 30 cm^2^. Thus, graphs of “1st pass and 2nd pass" of CFU/cm^2^ and microbial reduction (%) were constructed. Paired t-test was used to verify differences when the distribution was parametric and, for non-normal distributions, the Wilcoxon test was applied.

The standard deviation of the microbial log reduction (LR) was calculated as described by Pasternak^37^, where S_A_ and S_B_ refers to the sample standard deviations of the log reduction values for samples before and after test, respectively; and n_A_ and n_B_ refers to the number of replicates in the volunteers before and after treatment, respectively:

$${\text{SD}}_{{\text{LR}}}=\left[\left(\frac{S{a}^{2}}{nB}\right)+\left(\frac{S{b}^{2}}{nB}\right)\right]$$

Also, an additional analysis to better understand the results of the microbiological reduction effectiveness of the disinfection technology was applied. Principal Component Analysis (PCA) and construction of heat maps using ClustVis, an online tool for visualization of multivariate data clustering, were performed^38^. The Log_10_ average values ​​were used to construct two more analyses. The evaluated surfaces were applied in the four rows of the matrix, while the recruitment codes referring to the study participants were placed in the columns. In addition, two classifications were adopted for data analysis, applied to the columns of annotations: (1) means of transport used by the participant when traveling to the workplace; and (2) activity performed by the participant, being divided into “general services", encompassing functions that could theoretically expose the participant to greater environmental contamination, such as cleaning, gardening, maintenance and “other" services, involving participants from administrative areas and less exposure/circulation between environments. The purpose of these classifications was to observe the behavior of the relationship between the two classifications evaluated and verify whether the labor activity performed could influence the results of reduction effectiveness, considering a greater exposure to environmental contamination, as well as verifying the formation of clusters on the maps of heat.

For the PCA, the configurations of (1) singular value decomposition (SVD) were applied, with imputation used to calculate the principal components; (2) unitary variation scale applied to the lines; and (3) prediction ellipses, with a probability of 0.95, used to observe whether a group would fall within the probability ellipse of another group, or if they would distance themselves from each other. On the other hand, for the heat maps, the configurations were (1) centered lines; (2) unit variance scale applied to the lines; and (3) both rows and columns were grouped using the correlation distance and the mean link.

### Ethics approval and consent to participate

This study was conducted after the project was approved (Report No. 4.739.411) by the Research Ethics Committee (CEP) of the SENAI CIMATEC University Center (Orlando Gomes Avenue, 1845-Piatã, Salvador, State of Bahia, Brazil, Zip code 41650-010). In addition, we confirmed that informed consent was obtained from all participants.

## Results

To determine the antimicrobial action of the ozonized water sprayed by the disinfection chamber, we collected samples from different surfaces (30 cm^2^) of the individuals' garments over a period of 30 days at the beginning (morning, 1st pass before the start of the workday) and at end of working hours (afternoon, 2nd shift at the end of the working day). The number of viable cells in CFU/cm^2^ (Log_10_), the reduction in CFU/cm^2^ (Log_10_) and the microbial reductions (%) were recorded in this period.

Before passage through the chamber, different microbial recoveries were taken for the surfaces. The number of viable cells recovered ranged from 2.59 Log_10_ (accessories, 2nd pass) to 4.04 Log_10_ (boot, 2nd pass). The highest recoveries were found for the boot and shirt, probably because they are more exposed surfaces and likely to come into contact with other surfaces, the soil for example. In terms of reduction, all garments, except for accessories in the afternoon, showed a reduction in microbial growth of Log_10_ > 2. The microbial reduction remained above 60% among surfaces. However, lower values ​​were found for boots in the morning (55.94%), in comparison with the other garments evaluated. Despite the lower reduction rate found, demonstrated that after spraying ozonized water for 30 s, it was possible to note that there was no statistical difference in the reduction (p = 0.776) of the number of viable microorganisms on this garment. All results of antimicrobial action are described in Table 1.

**Table 1:** Microbiological evaluation of the surfaces of different participants' garments after using the disinfection technology.

Surface	Period	Exposure condition	Number of viable cells in CFU/cm^2^ (Log _10_) ± SD (CI 95%)	Reduction in CFU/cm^2^ (Log_10_) ± SD	Microbial reduction percentage (%) ± SD (CI 95%)
Mask	1st pass	Before	3.16 ± 0.42 (2.93–3.39)	2.40 ± 0.40	75.95 ± 29.90 (59.50–91.40)
		After	0.76 ± 0.99 (0.23–1.29)		
	2nd pass	Before	3.23 ± 0.76 (2.82–3.64)	2.26 ± 0.44	69.97 ± 21.50 (61.88–84.87)
		After	0.97 ± 0.90 (0.49–1.46)		
Shirt	1st pass	Before	3.64 ± 0.78 (3.22–4.06)	2.18 ± 0.88	59.90 ± 31.20 (46.80–80.10)
		After	1.46 ± 1.48 (0.66–2.24)		
	2nd pass	Before	3.70 ± 0.57 (3.40–4.00)	2.14 ± 0.59	57.84 ± 29.50 (44.37–55.81)
		After	1.56 ± 1.26 (0.88–2.23)		
Boot	1st pass	Before	3.79 ± 0.88 (3.31–4.26)	2.12 ± 0.67	55.94 ± 24.60 (43.13–69.42)
		After	1.67 ± 1.16 (1.06–2.29)		
	2nd pass	Before	4.04 ± 0.52 (3.77–4.32)	2.29 ± 0.68	56.69 ± 27.20 (44.32–73.34)
		After	1.75 ± 1.37 (1.02–2.49)		
Accessories	1st pass	Before	2.79 ± 0.52 (2.30–3.27)	2.47 ± 0.16	88.53 ± 19.60 (70.79–100.00)
		After	0.32 ± 0.54 (0.00–0.81)		
	2nd pass	Before	2.59 ± 1.29 (1.39–1.78)	1.72 ± 0.97	66.41 ± 36.70 (37.98–100.00)
		After	0.87 ± 1.32 (0.00–2.09)		

*SD* standard-deviation, *CI 95%* confidence interval of 95%.

**Figure 4:**
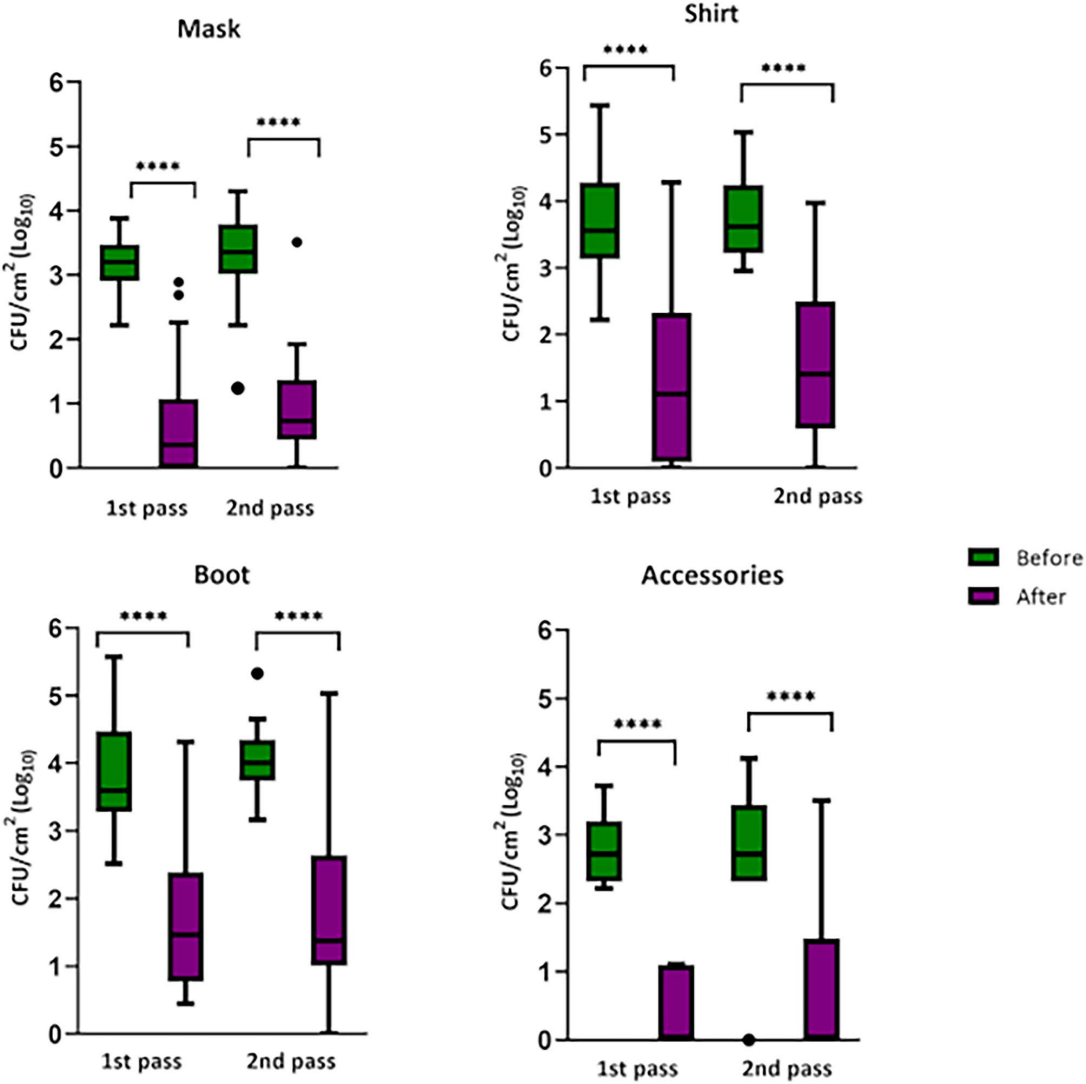
Comparison of the microbial growth of different garments in two periods of the day. In the morning, before the start of the working day (1st pass) and in the afternoon, after the working day (2nd pass); with 30 s pass time in disinfection technology. Accessories comprise the following objects: helmets, hats, watches and glasses. Individual points plotted beyond the box-and-whisker plot indicate values that extend to the minimum and the maximum. Asterisks indicate statistical difference after passing through the disinfection device (p < 0.0001) through the t Test and Wilcoxon Matched Pairs Test.

All surfaces tested showed a high number of viable cells before the first pass in the chamber (Log_10_ > 3) and a significant reduction (p < 0.0001, Test t and Wilcoxon test) after passing through the disinfection technology (Log_10_ < 1.75) within 30 s of exposure to ozonized water (Table 1; Fig. 4). The statistical differences found indicate that the results were significant from the statistical point of view, reflecting the reduction capacity brought about by the participants' passage through the disinfection chamber. This indicates that the observed data was not the result of chance, but rather attributed to the action of spraying ozonized water on the surfaces. Figures S2 and S3 presents the results of plating, growth and microbial reduction on the surfaces of some study participants. Furthermore, we observed that most of the CFU/cm^2^ (Log_10_) values that extended to the maximum and minimum values were found in the mask (Fig. 4, mask)**.**

**Figure 5:**
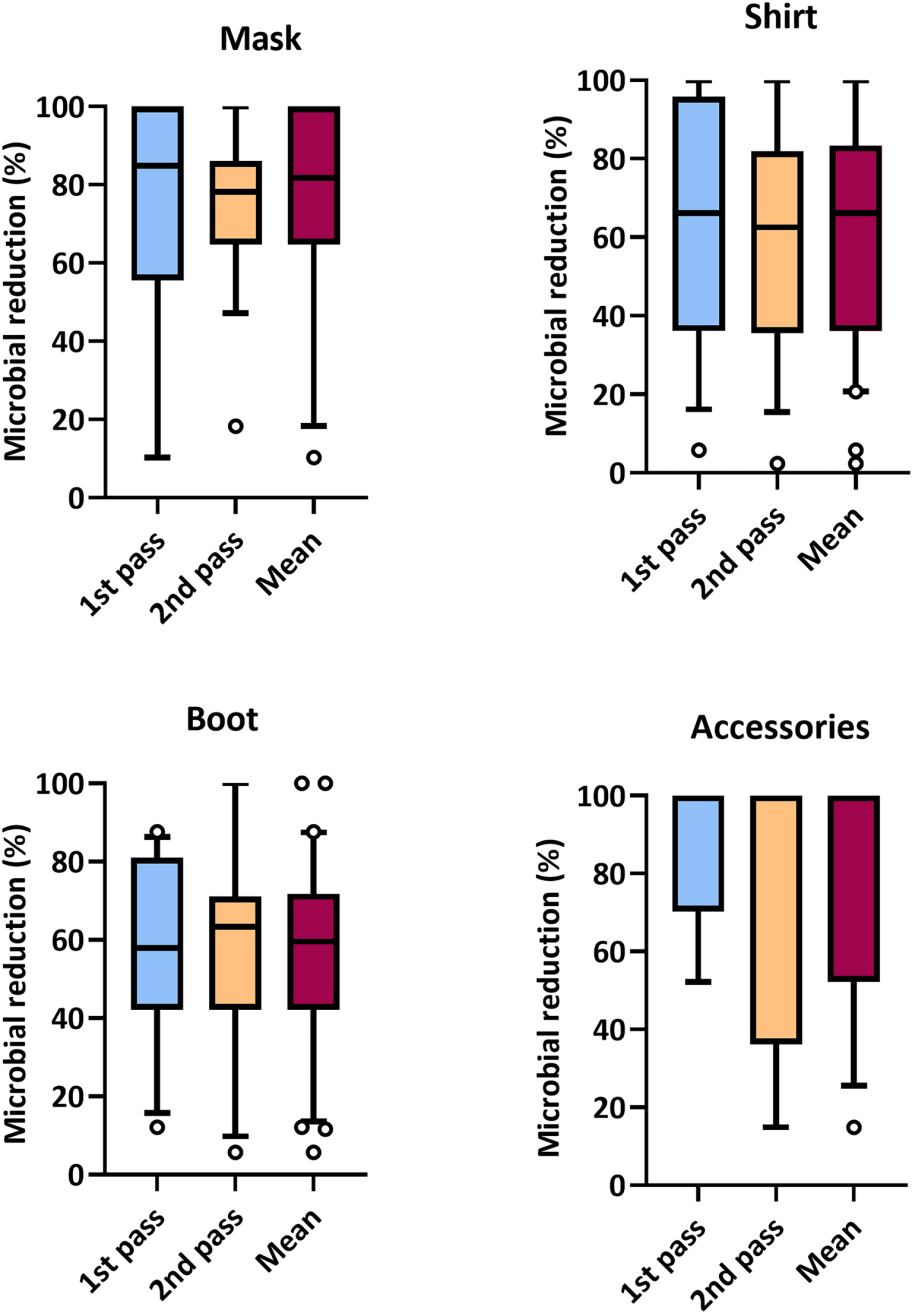
Comparison of the microbial reduction percentage (%) of different garments in two periods of the day. In the morning, before the start of the working day (1st pass) and in the afternoon, after the working day (2nd pass); with 30 s pass time in disinfection technology. Accessories comprise the following objects: helmets, hats, watches and glasses. Individual points plotted beyond the box-and-whisker plot indicate values that extend to the minimum and the maximum.

We also explored the difference in microbial reduction between times of day (morning and afternoon) for all surfaces through the reduction percentages (%). Microbial reduction percentage analysis found no significant difference using the Wilcoxon test for mask (p = 0.8040), shirt (p = 0.5698), accessories (p = 0.5000) and boots (p = 0.7760). The mask surface showed the highest rate of microbial reduction, presenting values of 75.95% and 69.97% for the 1st pass and 2nd pass, respectively (Table 1; Fig. 5, mask). For the other surfaces, such as accessories, satisfactory results were also found, with percentage averages above 80% of microbial reduction (Table 1; Fig. 5, accessories).

The analysis of PCA and heat maps were performed in order to obtain additional results to confirm the effectiveness of the disinfection technology, as well as to assess the influence of the two classifications used: (1) public or private means of transport and (2) function performed by the participant, being divided into “general services" (encompassing functions that theoretically could expose the participant to greater environmental contamination, such as cleaning, gardening, maintenance services) and “others" (involving participants from administrative and minor areas of exposure/circulation between environments). It is noteworthy that the classification of means of transport was only used for the generation of APCs and heat maps in the morning (1st pass), since after arriving at the work environment, contamination after carrying out the 1st pass would be associated with contamination by part of the workplace.

**Figure 6:**
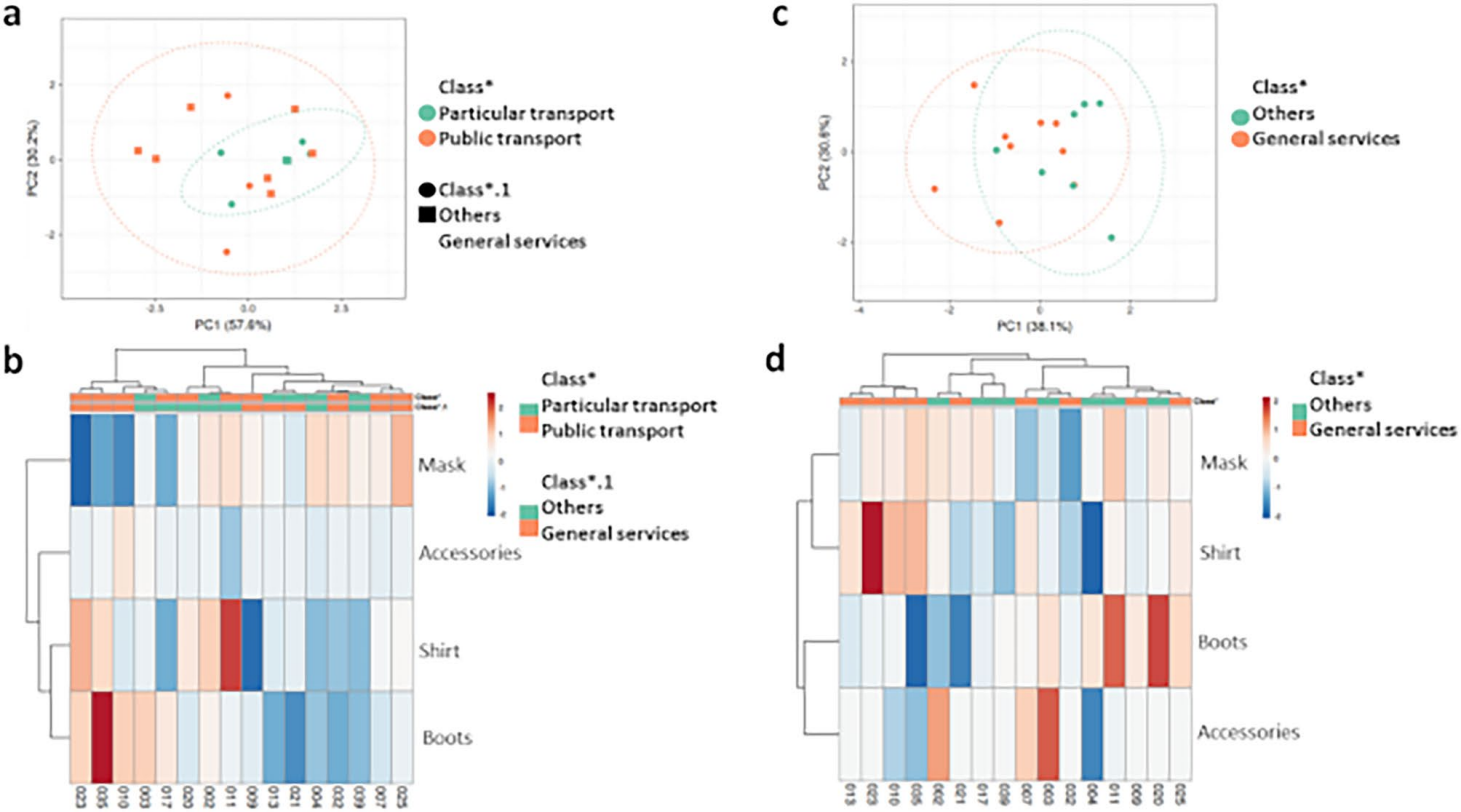
Confluent Log_10_ reduction analysis, after passing through the disinfection chamber. (**a**,**c**) PCA Graph for analysis of data referring to the 1st pass performed by the participants. No scale is applied to the lines and SVD with imputation is used to calculate the principal components. The X and Y axes show the main component 1 (PC1) and the main component 2 (PC2) which explain 57.6% and 30.2% of the total variation, respectively. Prediction ellipses were set to a probability of 0.95 and illustrate grouping by mode of transport and function classification, with indicated labels. (**b**) Heat map for data analysis referring to the 1st pass performed by the participants. The lines are centered and no scaling is applied to the lines. Imputation is used to estimate the missing value. Both rows and columns are grouped using the correlation distance and the average link. (**c**) PCA graph for data analysis referring to the 2nd pass performed by the participants. No scale is applied to the lines and SVD with imputation is used to calculate the principal components. The X and Y axes show the main component 1 (PC1) and the main component 2 (PC2) which explain 38.1% and 30.6% of the total variation, respectively. Analysis settings were the same as indicated in (**a**). (**d**) Heat map for data analysis referring to the 2nd pass performed by the participants. Analysis settings were the same as indicated in (**b**).

Figure 6 shows the average values ​​for the reduction in Log_10_ promoted by the disinfection chamber with spraying of ozonized water in the concentration range of 0.7–0.9 ppm, in the mornings (1st pass before the start of the journey of work) and in the afternoon (2nd passage after the working day). The result of the analysis showed the formation of two ellipses, where they end up overlapping (Fig. 6a,c). It is possible to observe that there was apparently no difference between the type of function performed by the participants who use private transport, only when the data were evaluated after the 1st pass (Fig. 6a), while for the data after the 2nd pass (Fig. 6b) the results are scattered among the classification ellipses. As for the means of transport, it is possible to observe that there was a difference for the group that uses public transport, with greater formation of ellipse and distance of data. Heat maps show the Euclidean distances between data, where similar data are darker in color. Based on Fig. 6b, it is possible to observe that the formation of clusters between the shirt and boot surfaces had an influence on the high values ​​of Log_10_ reduction in relation to participants 023, 035 and 010, users of public transport and embedded within the function of general services. It is possible to observe that these surfaces, for all participants, present this cluster formation further away from the mask surface, as well as from the accessories, indicating that despite some similarity between the reductions, it is not possible to globally compare the reduction values ​​obtained from the experiments performed, since they are not homogeneous in relation to the two classifications analyzed. The same can be said for the heat map analysis referring to the 2nd passage of the participants through the disinfection chamber, after the workday (Fig. 6d). In this case, however, the formation of a cluster took place between the accessory and boot surfaces; and between the shirt and mask surfaces, with a distance between these formations. It is noteworthy that the analysis for the surface of accessories was only performed on participants who have glasses, watches or helmets. Thus, in general, the results indicate that regardless of the level of contamination that surfaces may present, influenced or not by the means of transport used or exposure within work activities, the disinfection chamber is effective in promoting microbial reductions.

As for the analysis of the perception of use through the application of questionnaires to the study participants, of the total 40.6% (N = 43) were answered by female participants and 59.4% (N = 63) were answered by male participants. It is worth noting that confidentiality was assured to the participants. Furthermore, it is important to note that before use, the participants were provided with basic information about the disinfection technology and how it works, as well as for the biocidal agent used. Information mainly about biocompatibility of ozonized water was shared with them. In addition, the advantages and disadvantages were also clarified.

**Figure 7:**
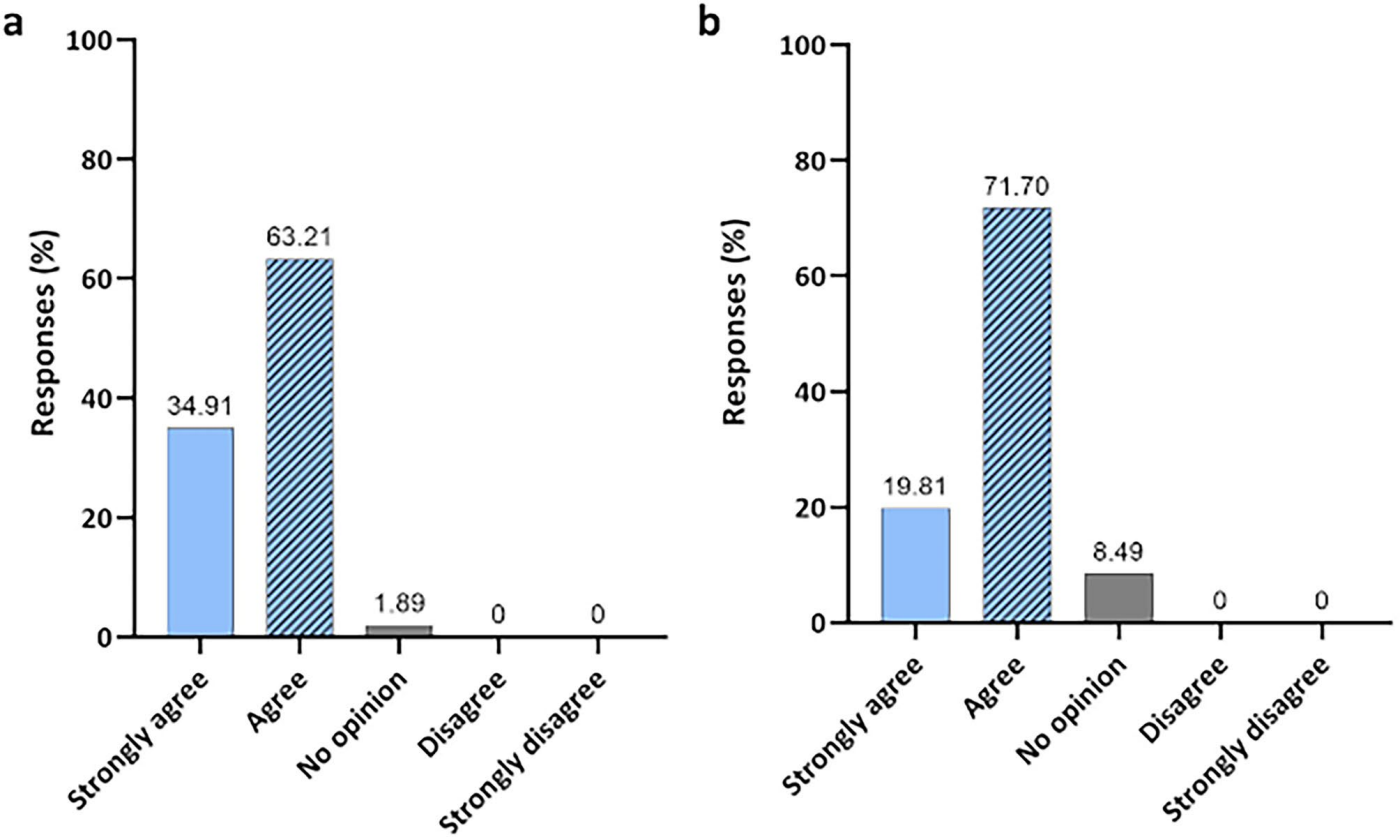
Participants' opinions. (**a**) Do you agree that ozonized water can be an alternative for disinfecting materials and surfaces, as it has an antimicrobial action?; and (**b**) ozone can be an effective alternative in controlling and spreading disease. Do you agree with this statement?

Figure 7 shows the results of two questions related to participants' familiarity with the biocidal agent used in disinfection technology. When asked whether they agreed that ozonized water could be an alternative for disinfecting materials and surfaces, due to the antimicrobial action presented by the agent, 34.91% (N = 37) and 63.21% (N = 67) responded strongly or I agree, respectively (Fig. 7a). They were also asked about O_3_, in its gas form, if they agreed with this agent to act effectively in the control and spread of diseases, in this case 19.81% (N = 21) strongly agreed, while 71.70% (N = 76) agreed with the statement (Fig. 7b).

**Figure 8:**
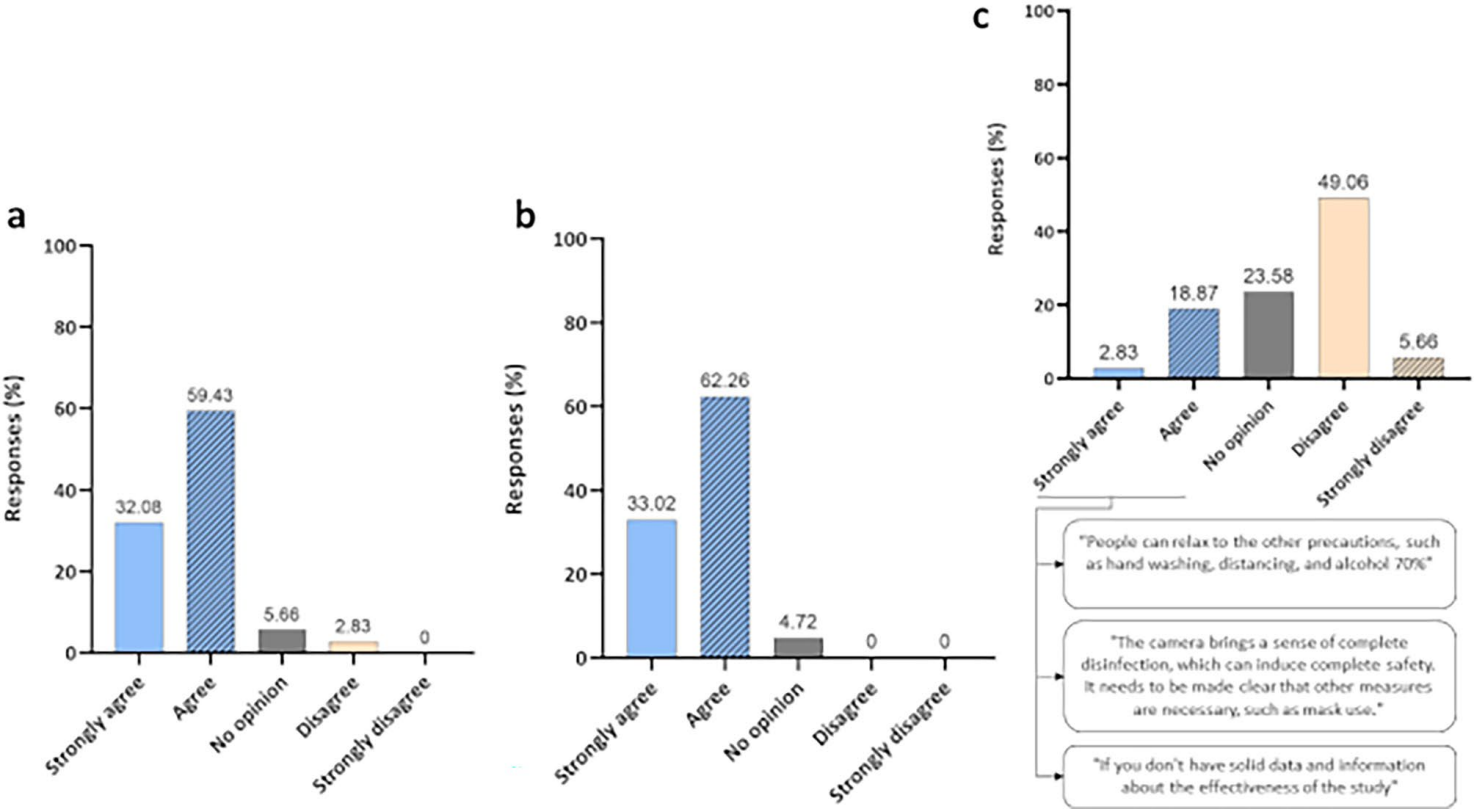
Participants' opinions. (**a**) After using the disinfection chamber, the surfaces were moistened; (**b**) the disinfection chamber acts as an additional barrier to the protection of workers in general; and (**c**) use of the disinfection chamber can lead to a false sense of security.

The next answers are related to perceptions regarding the use of the disinfection chamber. It is noteworthy that none of the participants reported feeling discomfort after using the technology, not showing any type of effect related to the disinfection chamber or the biocidal agent. Figure 8 presents the result regarding the three questions asked about the technology and the percentages of agreement or not of the participants in relation to them. A total of 32.08% (N = 34) and 59.43% (N = 63) strongly agreed or agreed, respectively, regarding the humidification of surfaces provided after the use of the disinfection chamber (Fig. 8a). Only 2.83% (N = 3) disagreed with the statement and 5.66% (N = 6) of the participants did not know how to give their opinion. As for the question about the disinfection chamber working as an additional protective barrier, 33.02% (N = 35) strongly agreed and 62.26% (N = 66) agreed. None of the participants disagreed with this statement, while only 4.72% (N = 5) did not know how to give an opinion (Fig. 8b).

When asked about the false sense of security that the use of the disinfection chamber could lead to, 49.06% (N = 52) of the participants disagreed and 5.66% (N = 6) strongly disagreed, while 2.83% (N = 3) and 18.87% (N = 20) strongly agreed or agreed, respectively, with the statement. 23.58% (N = 25) did not know how to give their opinion (Fig. 8c). In this case, for the participants who responded that they believed that technology could generate a false sense of security (total N = 23), they were asked to explain why. Most comments (Fig. 8c) were related to concerns about the neglect of other protective measures, such as hand hygiene and the use of protective masks. In addition, the low availability of scientific data that could prove the effectiveness of this type of technology was highlighted. In this regard, information about laboratory and initial field trials results obtained in previous experiments was shared with the participants. Although the availability of data is not yet widely disseminated, we have already found and reported that ozonized water under the physicochemical conditions of 4 °C and pH 5, resulted in high reduction percentages for *Staphylococcus aureus, Pseudomonas aeruginosa Enterococcus faecalis*, *Escherichia coli,* and *Candida albicans*. Images obtained from Scanning electron micrograph indicated that the effects on osmotic stability due to cell wall lysis might be one of the killing mechanisms of ozonized water. In addition, in assays using Hfib cells, the agent was biocompatible and presented no cytotoxic effect^32^. Before the use of disinfection technology by individuals, to confirm its biocidal action and verify the device’s efficacy, the reduction of the microbial load of important pathogens on PPE was also evaluated. The results showed that the instant decontamination system developed in this study proved effective for microbial reduction, confirming the potential of ozonized water as a biocidal agent^36^.

**Figure 9:**
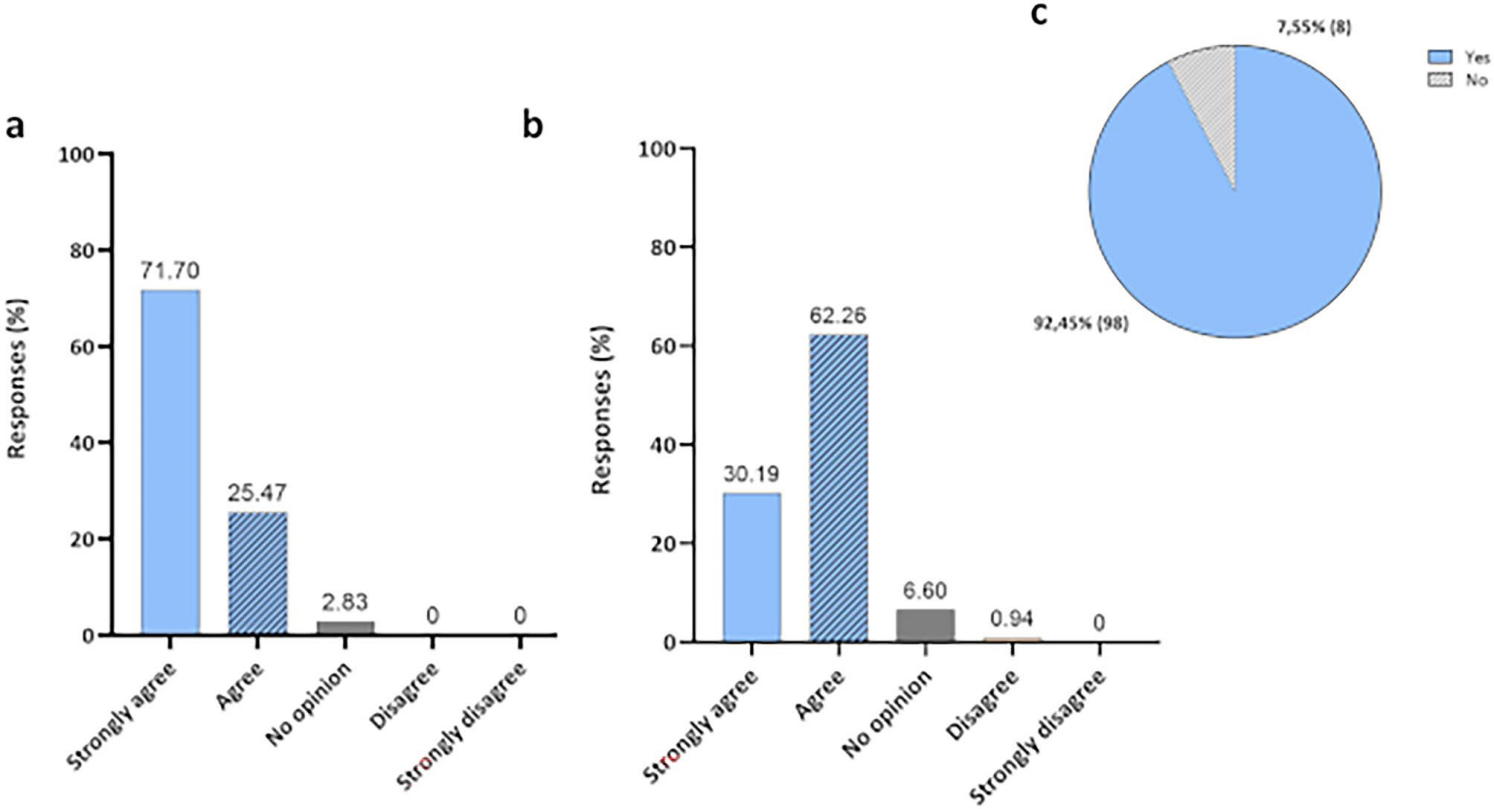
Participants' opinions. (**a**) Even with the use of the chamber, all personal hygiene procedures must be strictly followed; (**b**) the use of the disinfection chamber with ozonized water can be a safe alternative for surface disinfection; and (**c**) percentage approval of the disinfection technology used by study participants.

When asked about their agreement or not regarding the question that personal hygiene procedures must be strictly followed, even with the use of the chamber, 71.70% (N = 76) strongly agreed and 25.47% (N = 27) agreed with this statement (Fig. 9a). None of the participants disagreed, only 2.83% (N = 3) did not know how to give an opinion. Finally, 30.19% (N = 32) strongly agreed and 62.26% (N = 66) agreed that the use of the disinfection chamber with ozonized water can be a safe alternative for surface disinfection (Fig. 9b). This data reflects the 92.45% approval achieved in relation to the assessment of disinfection technology (Fig. 9c).

## Discussion

The aim of this study was to evaluate, through scientific evidence, the effectiveness of the disinfection technology developed, consisting of a disinfection chamber and an ozonation unit, through the use of ozonized water (0.7–0.9 ppm) as a biocidal agent, as well as bringing data about the perception of technology use by users of the device. The development of this technology was proposed to enable the use of a device capable of being used by people directly, with the spraying of ozonized water itself. In prospective studies focused on patents, unlike the decontamination objective of this technology, the vast majority of developed devices found are health applications and aimed at monitoring the cleaning processes of medical utensils and equipment, not focusing on emergency infection control. In addition, among the technologies coupled to an ozone generator, limited information is found about the stability of the product obtained, nor technical reports that guarantee effectiveness in reducing the general microbial load on different surfaces and for an adequate period of time after exposure to the biocidal agent; therefore, the effectiveness of these structures has not been clarified so far, which raises doubts to Organs health inspection bodies as to the real applicability of these products^39^.

In terms of advantages and disadvantages, each disinfection technology has unique characteristics and few studies are able to provide tangible data on costs and rarely on comparable costs (1 out of 43: 2%)^9^. The use of a certain type of disinfection technology must be determined by comprehensive consideration of economic and feasible factors such as the amount of wastewater, safety conditions, supply of disinfectants, investment and operating costs, level of management of the operation, etc. Chlorine and sodium hypochlorite, for example, are part of disinfection technologies with low investment and operational costs. However, they offer high storage risks, strong corrosiveness, high pollution and toxicity. On the other hand, even with a higher operational cost, the use of ozonized water does not generate environmental pollution and adverse effects on human health^32,40^, which is of great importance for the development of disinfection strategies.

In relation to microbiological tests and application of questionnaires, aspects that have so far been little addressed when referring to the evaluation of a fast and safe surface disinfection technology for use in humans, as currently the decontamination strategies have been tested using air treatment using O_3_ and relative humidity^41,42^ or vapor-based fumigant systems for disinfecting surfaces and environments^43^. Historical documentation reports that the first identification of O_3_ as a distinct chemical compound was made in water, which, after electrolysis, emanated a characteristic odor defined as “the odor of electrical matter", which was later defined as “Ozone", from Greek ozein (odorant)^44^. Furthermore, at the time it was already suggested that O_3_, as an oxidizing agent, could also be exploited as a strong disinfectant. This hypothesis was further validated in the late nineteenth century, when several reports showed the oxidation of organic compounds and the inactivation of bacterial contaminants in sewage after exposure to O_3_. Therefore, O_3_ was also proposed as an alternative treatment to water chlorination. Furthermore, it can be used for the treatment of potentially contaminated surfaces, water and ambient air, thanks to its powerful germicidal effect on a wide spectrum of microorganisms^45–47^.

The stability of O_3_ dissolved in water (ozonized water), from changes in physicochemical parameters, is essential for the agent to be considered a potent antimicrobial and useful in disinfection processes^48^. The physicochemical parameters adopted in our study, such as temperature between 4 and 7 °C and absence of water buffering, and the concentration of O_3_ (0.7–0.9 ppm), not showing cytotoxic effects on human cells, proved that ozonized water can be considered a viable alternative for microbial control^32^. Dhillon et al.^49^ also concluded that the quality of ozonized water (tap, distilled and ultrapure), temperature (7 °C) and pH 6.5 in a system is effective in reducing the microbial load in foods.

This concentration of O_3_ (0.7–0.9 ppm) dissolved in water was sufficient to eliminate a considerable number of microorganisms from the surface of the garments after 30 s of exposure. This result shows that the antimicrobial action of O_3_ is related to its powerful oxidizing action, which at relatively low concentrations of O_3_ and a short contact time are sufficient to inactivate several microorganisms^50,51^. The antimicrobial effect of ozonized water (0.1 ppm) has also been effective in reducing the total bacterial load of anaerobes (51.7%) and *Streptococcus* (56.4%) in supragingival plaque samples, after rinsing for 30 s^52^. On the other hand, Cesar et al*.*^53^ observed a microbial Log reduction dependent on exposure time (10 and 30 min) to ozonized water in *Escherichia coli* (2.72–3.78 Log), followed by *Staphylococcus aureus* (2.14–3.19 Log), *Candida albicans* (1.44–2.14 Log) and *Bacillus atrophaeus* spores (1.01–1.98 Log) in dental instruments.

We were able to analyze the colonization of the individuals' surfaces through the total count of mesophilic aerobics and total fungi in the two periods of the day, and we observed that there was a reestablishment of the number of viable cells at the end of the shift after a considerable reduction in CFU/cm^2^ in the first pass (start of working day). This result is similar to the study described by Vargas-Robles et al.^54^, that observed changes in the microbiome of volunteers during a subway ride, although hand washing immediately reduced biomass and diversity, traveling increased bacterial diversity to the same levels that after traveling when there was no hand washing procedure. Likewise, we believe that after the 1st passage in the disinfection chamber, the beginning of work activities has contributed to the microbial reestablishment in the garments of the individuals in this study.

It is important to highlight that the fact that the microorganisms re-establish themselves at the end of the working day indicates that the disinfection technology is effective in an 8-h workday, since even with the microbial reestablishment during the day, a microbial Log reduction was also observed after the 2nd passage through the disinfection chamber (at the end of the working day). This information is corroborated by the results found in the PCA and heat map analyses, which demonstrated that regardless of the level of exposure and contamination acquired during the workday, the biocidal effect of ozonized water occurs regardless of the type of surface evaluated or index of initial contamination. After decontaminating floors, toilet seats and soap dishes in four public restrooms, Gibbons et al.^55^ also tracked microbial colonization on bathroom floors and observed development within 5–8 h of a successional community with remarkable stability over weeks and months. Associated human microbiota, including *Staphylococcus* strains, can remain viable on these surfaces for many hours after their dispersal agents have been removed and could be significant fomites for viable human pathogens.

Perception analysis for technology evaluation based on the application of questionnaires is an important tool in complementing the data on the effectiveness of the developed technology. Similar studies to evaluate other disinfection devices were also carried out. Rock et al.^56^ conducted research to investigate the impact of ultraviolet UV-C disinfection applied to the elimination of healthcare-associated pathogens in patient rooms of an academic hospital. Dunn et al*.*^57^ also used the same methodology for applying questionnaires for a survey about the use of a device also with application of UV-C radiation for decontamination of rooms. Although specific evaluation studies for disinfection chambers and their direct use by people were not found, these studies show that the use of questionnaires to understand the perceptions and acceptance of individuals in relation to the development of new technologies is important in coping with the challenges involved, especially when it comes to the analysis of a new biocidal agent applied for the first time in this case. Within this context, given the results obtained regarding the absence of discomfort when using the disinfection chamber of this study, this data confirms the reduction of sensations generated in relation to O_3_ in its gas form, when dissolved in water^27,58,59^. Furthermore, this feat may be related to the concentration used, not exceeding sensory limits for the participants.

It is noteworthy that limitations could be observed regarding the humidification of surfaces after passing through the disinfection chamber, influencing the results of microbial reduction. This indicates that the human factor may contribute to the (in)correct distribution of the biocidal agent across surfaces, as assessed by Neves et al*.*^35^, as it depends on the correct implementation of the technology's instructions for use; and other considerations to be taken into account, such as the differences between the materials/fabrics that make up the different surfaces.

In addition, as claimed by the participants of this study, regulatory agencies have already warned about the false sense of security that can result from the use of this type of device^60^, however we emphasize that this technology aims to act in conjunction with other actions of proven effectiveness, such as social distancing and the use of a mask (in the specific case of COVID-19) and hygiene measures in general, such as hand washing, which do not discourage such measures. Accordingly, 97.17% of the participants in this study reinforce the understanding of the need to maintain these other prevention procedures, believing that the disinfection chamber with ozonized water spray acts as an additional barrier contributing to help protect and reduce contamination of surfaces.

Another highlight is the need for scientific proof necessary to give reliability as to the microbial reduction capacity, as well as security for users of these types of technologies. As for the biocidal agent, sufficient scientific data can be found in the literature attesting to the antimicrobial capacity of ozonized water against viruses, fungi and bacteria^28,32,61–64^ and its application even in clinical procedures that do not lead to cytotoxicity or toxicity to users^32,65–69^.

With the advent of the pandemic, surface disinfection became essential, being included in several national and international policies and recommendations^12,70,71^. This process do not include only the decontamination of areas, but supplies such as masks, that take place because of the negative health impact from an insufficient supply of N95/FFP2 masks for healthcare workers^72,73^. In contrast to hand hygiene, a daily activity performed by people, the view that environmental disinfection is important, and methodologies and technologies that can support this process have recently begun to gain ground^74^, not only for the COVID-19 situation, but also for other contaminations. However, the lack of scientific data associating the use of this biocidal agent (or others) in disinfection technologies for use by individuals makes the authorities' concern about the dissemination of these devices valid.

We have recently provided scientific evidence of the full and partial biocidal effect on PPE contaminated by human pathogens of medical importance and public health impacts, namely *Staphylococcus aureus*, *S. epidermidis, Enterococcus faecalis*, *Escherichia coli*, *Pseudomonas aeruginosa*, *Serratia marcescens, Citrobacter freundii*, *Proteus mirabilis*, *Candida albicans* and *Candida parapsilosis*, with microbial log reductions above 2 and reduction percentages above 60% (0.3–0.6 mg/L O_3_) and 80% (0.7–0.9 mg/L of O_3_), with a high proportion of the tested PPE showing 100% microbial reduction. In addition, viral inactivation in a Gammacoronavirus model was evaluated, as a way to mimic the action of this agent against SARS-CoV-2. Inactivation above 99% was proven through in vitro tests performed. These results show the potential for the use of this biocidal agent, which can positively and safely contribute to the containment and control of microbial infections in humans^36^, including being able to be used in emergency situations, such as the COVID-19 pandemic.

Hence, as a way to continue the effectiveness analysis, the purpose of developing a technology that can be used as a protection barrier is highlighted, precisely to seek the evaluation of this technology developed (which obtained 92.45% approval by the research participants) and scientific contribution in relation to the data found, ensuring its safe use and capable of being used in contexts of outbreaks or even in routine disinfection procedures in places with high incidence and/or dissemination of pathogens of medical importance, in support of the protection measures already implemented.

## Conclusion

The disinfection technology developed, comprising the disinfection chamber and the ozonation unit, showed relevant results in terms of efficacy regarding the use of the biocide agent ozonized water at a concentration of 0.7–0.9 ppm. Microbiological tests confirmed the ability to promote the microbial reduction of total bacteria and fungi with high reduction percentages, exerted by passing through the disinfection chamber for a time of up to 30 s. Furthermore, from the analysis of the colonization of surfaces in the two periods of the day (1st and 2nd passage), it was possible to demonstrate the effectiveness of reduction regardless of microbial restoration through exposure to environmental contamination. Finally, the analysis of the understanding of the participants' perceptions and acceptance in relation to the use of disinfection technology contributed positively to reinforce the need for the development and use of new technologies that can help fight the spread of infectious agents, helping to fight diseases disseminated through surface contamination.

## Supplementary Information

Supplementary Information.

## Data Availability

All the results found are available in this manuscript.
