# Supplementary material for: Evaluation of the microbial reduction efficacy and perception of use of an ozonized water spray disinfection technology
Source: Sci Rep. 2022 Jul 29;12:13019. doi: 10.1038/s41598-022-16953-2 (PMC9335460; doi:10.1038/s41598-022-16953-2)
Supplement: Supplementary file 1 — Supplementary Information. [file 41598_2022_16953_MOESM1_ESM.pdf]

Supplementary information for

Evaluation of the microbial reduction efficacy and perception of use of an ozonized water spray disinfection technology

Luis Alberto Brêda Mascarenhas[1], Laerte Marlon Conceição dos Santos[1], Fabricia Oliveira Oliveira[1], Leticia de Alencar Pereira Rodrigues[1], Paulo Roberto Freitas Neves[2], Greta Almeida Fernandes Moreira[1], Alex Alisson Bandeira Santos[2], Gabriela Monteiro Lobato[3], Carlos Nascimento[3], Marcelo Gerhardt[3] and Bruna Aparecida Souza Machado[1*]

[1]University Center SENAI/CIMATEC, SENAI Institute of Innovation (ISI) in Health Advanced Systems (CIMATEC ISI SAS), Salvador 41650-010, Bahia, Brazil;

[2]University Center SENAI/CIMATEC, SENAI Computational Modeling and Industrial Technology, 41650-010, Salvador, Bahia, Brazil.

[3]China Three Gorges Corporation – CTG Brazil, Rio Paraná Energia S.A. Rodovia MS-444 s/nº km 58, Ilha Solteira, Selviria (MS), Brazil;

*Corresponding Author

E-mail: brunam@fieb.org.br (BASM)

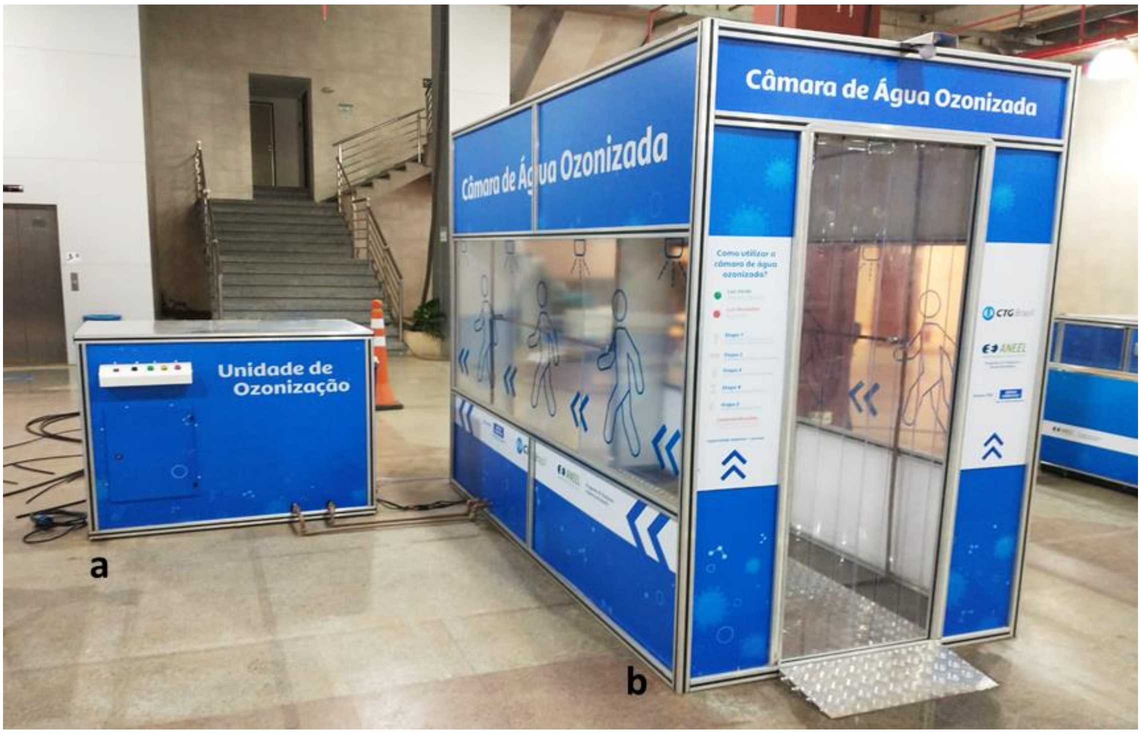

Fig S1. Developed disinfection technology composed of an (a) ozonation unit, responsible for producing ozonated water at a concentration of 0.7 to 0.9 ppm and an (b) ozonized water spray disinfection chamber.

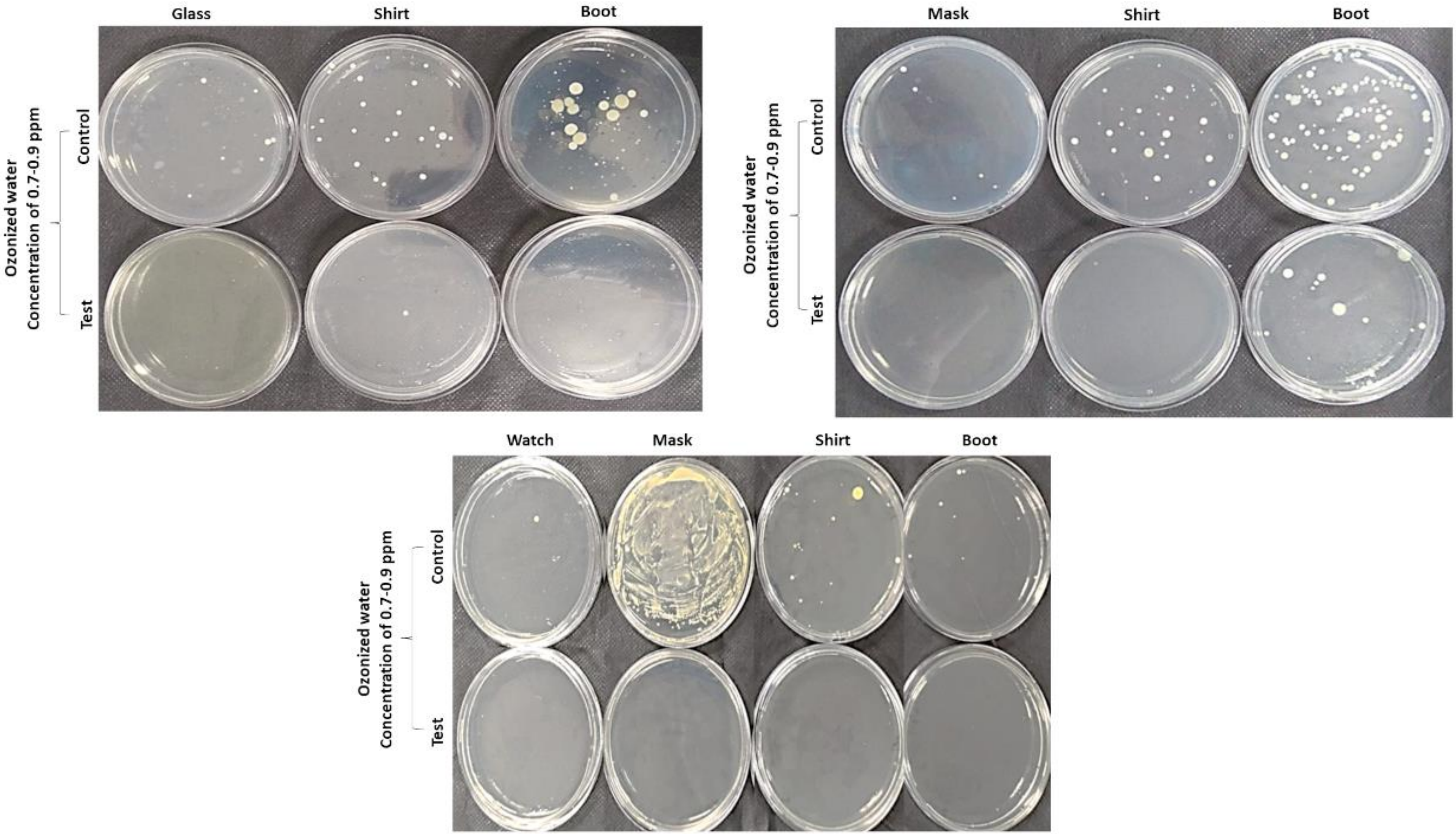

Fig S2. Representative plating images of the contamination recovered from the evaluated surfaces before and after passing through the disinfection chamber, of different participants of the study.

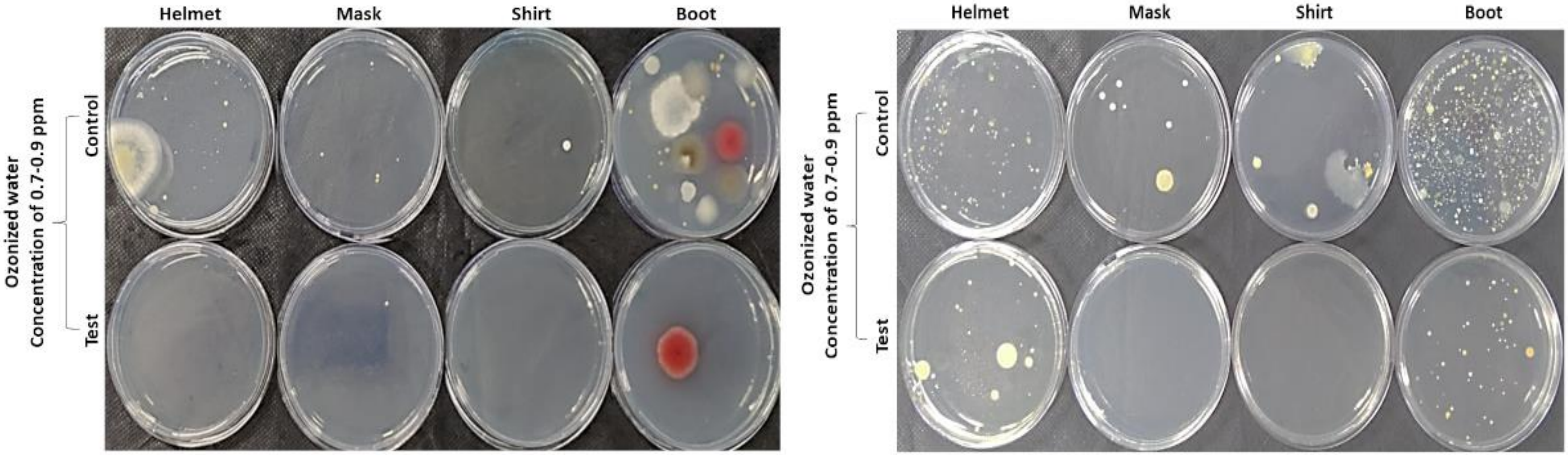

Fig S3. Representative plating images of the contamination recovered from the evaluated surfaces before and after passing through the disinfection chamber, of different participants of the study.
